# Identifying the key factors of subsidiary supervision and management using an innovative hybrid architecture in a big data environment

**DOI:** 10.1186/s40854-020-00219-9

**Published:** 2021-02-04

**Authors:** Kuang-Hua Hu, Ming-Fu Hsu, Fu-Hsiang Chen, Mu-Ziyun Liu

**Affiliations:** 1 School of Accounting, Finance and Accounting Research Center, Nanfang College of Sun Yat-Sen University, Guangzhou, China; 2 English Program of Global Business, Chinese Culture University, Taipei, Taiwan; 3 Department and Graduate School of Accounting, Chinese Culture University, Taipei, Taiwan; 4 School of Accounting, Nanfang College of Sun Yat-Sen University, Guangzhou, China

**Keywords:** Decision making, Swarm intelligence, Internal audit, Fuzzy rough set theory

## Abstract

In a highly intertwined and connected business environment, globalized layout planning can be an effective way for enterprises to expand their market. Nevertheless, conflicts and contradictions always exist between parent and subsidiary enterprises; if they are in different countries, these conflicts can become especially problematic. Internal control systems for subsidiary supervision and management seem to be particularly important when aiming to align subsidiaries’ decisions with parent enterprises’ strategic intentions, and such systems undoubtedly involve numerous criteria/dimensions. An effective tool is urgently needed to clarify the relevant issues and discern the cause-and-effect relationships among them in these conflicts. Traditional statistical approaches cannot fully explain these situations due to the complexity and invisibility of the criteria/dimensions; thus, the fuzzy rough set theory (FRST), with its superior data exploration ability and impreciseness tolerance, can be considered to adequately address the complexities. Motivated by efficient integrated systems, aggregating multiple dissimilar systems’ outputs and converting them into a consensus result can be useful for realizing outstanding performances. Based on this concept, we insert selected criteria/dimensions via FRST into DEMATEL to identify and analyze the dependency and feedback relations among variables of parent/subsidiary gaps and conflicts. The results present the improvement priorities based on their magnitude of impact, in the following order: organizational control structure, business and financial information system management, major financial management, business strategy management, construction of a management system, and integrated audit management. Managers can consider the potential implications herein when formulating future targeted policies to improve subsidiary supervision and strengthen overall corporate governance.

## Introduction

With globalization and the rise of emerging economies, many companies have simultaneously invested in domestic and foreign countries and set up subsidiaries to promote rapid growth by developing more markets and enhancing market competitiveness (Decreton et al. 2019; Doz et al. 2001). Faced with existing political and economic environments and market changes in different regions while trying to avoid the contradiction of business diversification, firms must simultaneously ensure that subsidiary operations do not deviate from the parent company’s strategic intentions and harm its investment interests (Hensmans and Liu 2018; Stea et al. 2015). To address the problem of subsidiary management, Chiang et al. (2008) investigated the relationship between the parent firm and subsidiaries required to reach the goal of smooth operations and positive performance outcomes. Strengthening subsidiary supervision is a major problem for parent companies when they take actions to expand their business success by adding overseas operations (Nuruzzaman et al. 2018).

Expansion by parent companies is important to national economic development; therefore, establishing a scientific supervision system for subsidiaries is crucial for the survival and development of these companies (Zhao et al. 2013). When an enterprise sets up subsidiaries, it needs to keep pace with the market and continuously adjust its business strategies. Furthermore, industry characteristics and both internal and external environments must be considered when establishing supervision to effectively prevent and resolve various risks and obtain competitive advantages. As a gatekeeper of a parent company’s supervision over its subsidiaries, an internal audit is paramount to any management success (Cahill 2006). It can supervise the entire process of subsidiaries’ business activities and transfer internal information through multiple channels in order to reduce the asymmetry of an enterprise’s internal information and to realize comprehensive subsidiary monitoring. Similarly, as an important component of corporate governance, an internal audit helps the parent company conduct more effective subsidiary supervision by identifying the risks subsidiaries may face in ever-changing environments (Zaharia et al. 2014; Arthur 2017).

An internal audit evaluates the level of enterprise risk management and the effectiveness of internal control, helps a firm achieve strategic goals, and adds value to operations by providing suggestions for enterprise management (Gul et al. 2018). An effective internal audit can ensure the quality of subsidiary supervision, assist the parent company’s management, and improve internal controls. Based on the goal of effectively performing their fiduciary responsibility, an internal auditor participates in corporate governance activities by evaluating the internal controls of various specialized areas within an organization, thus forming a balance of power (Singh 2003). In this way, an internal audit can go beyond the traditional role of monitoring internal control procedures and play a special role in governance. In this role, an internal audit improves the quality of subsidiary governance, aids in achieving the parent company’s goals, and helps managers better arrange and more effectively control subsidiary operating activities. Thus, it is necessary for businesses to clarify the required factors for subsidiary supervision and management.

Prior studies on subsidiary supervision and management are not comprehensive and primarily consist of empirical case studies (Botez 2012; Petrascu and Tieanu 2014) or regression analysis (Jaussaud and Schaaper 2006; Luo 2001). However, the assumptions of independence, normality, and linear relationships between variables in these traditional statistical methods are often inconsistent with the real-world characteristics of the complex and intertwined relationships among supervision elements, limiting the ability to extract important information. Accordingly, to comprehensively explain real-world situations and explore hidden information, numerous artificial intelligence (AI)-based approaches have been introduced to handle internal control issues, such as performance measurement, (Dossi and Patelli 2008; Fitzgerald and Rowley 2015), credit rating analysis (Yu et al. 2015), mutual fund performance (Kong et al. 2019; Moradi and Mokhatab Rafiei 2019), and corporate governance (Alharbi et al. 2016), without satisfying strict statistical assumptions.

For unknown domains, users generally want to collect as much information as possible to understand the underlying situation. However, too much information confuses users and leads to biased judgments. To handle this challenge, data exploration that aims to assist users in realizing the investigated reality faster and provide reliable results. The rough set theory (RST) (Pawlak 1982) is a potent mathematical tool for data exploration. Use of RST allows for the exploration of data via a rule representation structure, such as an if_(condition)-then_(decision) representation, which makes it possible to conveniently depict a knowledge domain as relations connecting premises and conclusions arising out from the observations of those premises (Nowak-Brzezińska and Wakulicz-Deja 2019). Furthermore, if the extracted knowledge cannot be examined or tested by users, they will have a strong incentive *not* to employ this model and to impede its practical applications. The knowledge expression in an if–then format has demonstrated its intuitiveness and clearness. Thus, RST has been widely initiated upon many research domains, such as quality prediction (Yin et al. 2019), energy consumption (Cao et al. 2020), spam classification (Dutta et al. 2018), stock exchange (Joulaei and Mirbolouki 2020), and customer churn prediction (Vijaya and Sivasankar 2018).

Traditional RST has two significant data pre-processing deficiencies that need to be overcome: (1) it only can handle crisp data (i.e., it cannot handle real-valued data) and (2) it is inadequate at finding the minimal reduct (i.e., the smallest sets of features possible) because its run time of generating all reducts is exponential (Chang et al. 2018; Keramati et al. 2016; Jensen and Shen 2005). To solve these issues simultaneously, this study adopted the characteristics of the membership function of the fuzzy set theory (FST) to solve the RST defect of data loss and to improve data quality and the reliability of decision-making. By integrating FST into RST, the hybrid model (i.e., fuzzy rough set theory, FRST) can handle both data structures (i.e., crisp data and real-valued data) and also can extend their practical applications (Jensen and Shen 2005).

An additional problem with traditional RST is finding the best variable combination (that is, the minimal reduct). In past decades, direct calculations (i.e., greedy search) were used to search for the best solutions, but this technique consumes considerable time and resources and easily falls into a local optimal (Jensen and Mac Parthaláin 2015; Lin et al. 2019; Chang and Hsu 2019). Recently, many scholars have proposed adapting the concept of swarm intelligence, which is based on observations of the behavior of natural creatures, and the ant colony optimization (ACO) algorithm, as it can best highlight its characteristics (Paul and Das 2015; Shunmugapriya and Kanmani 2017). The ACO approach simulates ants’ pheromone scattering on search paths while they forage for food; they search for the best path through the continuous accumulation of pheromones and then seek the best solution through iteration. Compared to a genetic algorithm (GA) or greedy search, ACO, with its greater flexibility and superior performance (Jensen and Shen 2005), has been successfully applied to a large number of different combinational problems, such as warehouse management (Arnaout et al. 2020), product usability (Midhunchakkaravarthy and SelvaBrunda 2020), ecological emission (Raviprabakaran and Subramanian 2018), and energy management (Sutar et al. 2020). Additionally, combining multiple system outcomes and translating them into one conclusive result can lead to outstanding performance. The fundamental idea is to complement any error(s) made by a singular system. Therefore, the joint utilization of ACO and FRST (ACO-FRST) complements their respective advantages—identifying the most essential features for users to gain more insights plus reducing computational cost—and minimize disadvantages—determining the optimal reduct is time-consuming—echoing the “integrated systems” trend (Jensen and Shen 2005; Wang et al. 2007).

After performing ACO-FRST to explore the data, the selected criteria/dimensions are then fed into DEMATEL to reveal the interrelated and intertwined relations among evaluation criteria. Apart from prior statistical approaches, this method further considers the dependency and feedback relations among criteria (Ou Yang et al. 2013; Hu et al. 2017; Liu et al. 2018), allowing users to realize the influential directions and weights of the adopted criteria when forming a final decision. Abdullah and Zulkifli (2019) and Lin et al. (2020a, b) performed DEMATEL to illustrate relationships among the assessment criteria and filter out irrelevant and less essential attributes to prevent information overload. Asgaria and Abbasi (2015) also stated that the solution for a pairwise comparison in DEMATEL is very complicated for reaching a stable result if the dataset is too large. Özkan and İnal (2014) and Mohaghari et al. (2014) also indicated that it is hard to find an optimal solution when too many criteria/dimensions are considered simultaneously.

The purpose of this research is to provide a new approach to allow companies to manage their subsidiaries to maintain consistent internal control and align strategic goals using integrated analysis of management factors and recognizing unique risks faced by subsidiaries in ever-changing environments. We first link FST and RST for decision-makers to handle data with mixed types (i.e., crisp data and real-valued data) to reveal more intrinsic information for internal auditors. Next, the key factors screened by FRST are then fed into DEMATEL to depict the interrelated and intertwined relations among adopted criteria and to further examine their impact on the final decision. Next, we adopt interactive influential network relationship map (IINRM) derived from DEMATEL to realize which part of subsidiary supervision and management should be modified first to produce the most effective response. The results may help managers formulate firm policies to reach the company’s goals for sustainable development.

The remainder of this article is organized as follows. Section 2 describes the dimensions and criteria of supervision and management for subsidiaries in the existing literature. Section 3 describes the introduced hybrid model, while Sect. 4 analyzes the empirical results. Section 5 presents the discussion and practical application. Section 6 concludes.

## Supervision and management matrix

When the scale of subsidiaries continues to expand, regulatory issues between parent and subsidiary companies are gradually highlighted. Parent company supervision is crucial to the healthy development of subsidiaries, but in the current economic situation following the first several months of the COVID-19 pandemic, the effectiveness of subsidiary control does not meet expectations. As an independent and objective supervision, evaluation, and consulting activity, an internal audit can effectively supervise subsidiary production and operation, risk management, and governance structure, thereby improving subsidiary operating environments, promoting improvements in the corporate governance of parent-subsidiary companies, increasing corporate value, and achieving strategic objectives. Therefore, to help internal auditors clarify the key factors of subsidiary supervision and management based on the relevant literature (Ackermann and Fourie 2013; Ke 2018; Kostova et al. 2015; Markus and Martin 2019; Situmoranga and Japutra 2019; Zhao et al. 2013) and interviews with domain experts, the evaluation framework has been determined to include six dimensions: organizational control structure, business strategy management, construction of a management system, major financial management, business and financial information system management, and integrated audit management and its related sub-factors. The six dimensions and 31 criteria are identified in the second stage, as shown in Table 1.

**Table 1:** Criteria of supervision and management of subsidiaries for the pre-test questionnaire

Dimensions	Criteria
A: Organizational control structure	(a1) Directors and supervisors of subsidiaries
	(a2) Department division of responsibilities in subsidiaries
	(a3) Establishment of internal audit department
	(a4) Managers of subsidiaries
B: Business strategy management	(b1) Management of subsidiary operating efficiency
	(b2) Implementation of the subsidiaries' annual business plan
	(b3) Risk management of subsidiaries
	(b4) Strategic management of subsidiary development
C: Construction of a management system	(c1) Management of budget and final accounts
	(c2) Payment management
	(c3) Purchasing and supply management
	(c4) Production and inventory management
	(c5) Supplier management
	(c6) Information disclosure management
D: Major financial management	(d1) Operations management
	(d2) Investment management
	(d3) Capital Management
	(d4) Financing management
	(d5) Fixed assets management
E: Business and financial information system management	(e1) Financial and business communication system
	(e2) Provision of management reports
	(e3) Management security of information system
	(e4) Financial information system
F: Integrated audit management	(f1) Construction of internal control system in subsidiaries
	(f2) Implementation of annual internal audit plan
	(f3) Project audits of subsidiaries
	(f4) Rectification of internal audit issues
	(f5) Construction of internal audit teams in subsidiaries
	(f6) Perform internal audit of subsidiaries regularly or irregularly
	(f7) Internal audit quality control
	(f8) Competence of internal auditors in subsidiaries

### Organizational control structure

The organizational structure, a product of economic constraints imposed by environmental factors, is a system that clearly defines the division of labor and the rights and responsibilities in an organization; it affects corporate performance and financial decision-making (Child 1972; Lai and Limpaphayom 2010). Organizational structure is necessary to set up internal institutions properly, divide responsibility and authority scientifically, and promote business strategy implementation through cross-functional collaboration to ensure an enterprise operates smoothly in dynamic environments. Ke (2018) pointed out that the reasonable allocation of directors and supervisors of subsidiaries is a key means of strengthening subsidiary control. A board of directors can conduct operational control, strategic guidance, and business coordination through the management process (White 1979). When a parent company assigns board members and auditors to subsidiaries, subsidiary business strategy and business decisions can better cater to the parent’s interests, resulting in a more significant supervision effect (Cai et al. 2018). When the enterprise expands further, subsidiary corporations should reposition and redesign their management functions, stipulate the authority and responsibility of each department, and ensure coordination with the parent’s functional departments, thus improving the efficiency of subsidiary supervision (Boussebaa 2015).

### Business strategy management

Business strategy management provides long-term planning for overall enterprise development issues (such as production and operations management, marketing management, financial decision-making, risk management). A business strategy is influenced by the business’s complexity and the external environment’s uncertainty, but it also determines the company’s product structure and competitive market, technology, and organizational structures (Lim et al. 2018). On one hand, to achieve the synergy of strategy and operations in a parent-subsidiary company, the parent company imposes its overall strategic objectives into each subsidiary, then forms the subsidiary’s business objectives and plans, conducting scientific assessments of each subsidiary’s accomplishment of its annual business plan, thus strengthening management’s control over subsidiaries (Matolcsy and Wakefield 2017). On the other hand, the parent company needs to regularly evaluate the subsidiary’s operations, first pre-setting the subsidiary’s operating efficiency indicators (such as sales, profits, inventory), and then using the subsidiary’s financial reports to evaluate its annual operating benefits. Accordingly, the parent assesses the performance of the subsidiary directors and senior executives to achieve effective supervision of subsidiary businesses and finances.

### Construction of a management system

A management control system is the institutional arrangement for the daily business activities of enterprises, and it plays an important role in the management of the business by guaranteeing the execution of corporate strategies (Matolcsy and Wakefield 2017). In the process of supervising subsidiaries, the institutional system and operational mechanisms established by the parent company regulate various aspects of the subsidiary’s daily operations. Management of the budget and final accounts is an important means of planning and control in enterprise management (Dunk 2001; Defranco and Schmidgall 2017; Djebali and Zaghdoudi 2020) to provide a basis for the parent company to supervise its subsidiaries and conduct business planning and performance evaluations (Markus and Martin 2019). Also, Sonia et al. (2014) found that working capital management (such as cash management, accounts payable management, accounts receivable management) has a positive impact on corporate financial performance, and payment management plays a significant role in adjusting corporate cash flow and improving the benefits of working capital management. Otherwise, a scientific procurement management system will significantly improve the efficiency and effectiveness of enterprise operations (Weele and Raaij 2014). If the parent company continues to expand, establishing an effective procurement management system will help achieve cost savings and resource optimization, and enhance subsidiaries’ continued competitiveness (Johnsen 2018).

### Major financial management

As the number of subsidiaries continues to increase, the parent company faces a more complex and more volatile environment, and the challenges of corporate strategic management, organizational operations, and capital management follow (Gibbons et al. 2011; Schotten and Morais 2019). Operations management is a key factor in an enterprise’s survival (Rahiminezhad Galankashi et al. 2020). By transforming enterprise investment into output, operations management can gain a competitive advantage in the market and help enterprises realize value-added (Bromiley and Rau 2015). Cheng et al. (2013) point out that the quality (return on assets) and efficiency (capital recovery period) of an investment directly affect corporate financial performance, because the risk in enterprise investment is not easy to control in such a complex procedure. Therefore, the parent company needs to pay attention to the investment behavior of its subsidiaries to avoid their deviation from the parent company’s overall goal, thus affecting the company’s financial indicators and business performance (Chang and Taylor 1999). Capital management is considered an important contributor to the creation of corporate value (Mortensen 2014). Some studies have also found that the parent company can improve overall profitability through effective subsidiary working capital management (Büyüközkan and Güler 2020; Yue 2019).

### Business and financial information system management

In view of the role of information production, delivery systems, and communication coordination mechanisms in supervision and management, how the financial department uses the information system to realize business coordination and financial integration between the parent company and its subsidiary is a key supervision issue. Consistency between the enterprise information system and business strategy has a certain effect on the achievement of organizational goals. Sharing and integrating financial and business data facilitates the communication channels between parent companies and subsidiaries (Rao 2012). Internal information systems can provide accurate and timely business and financial information for senior management, and enhance corporate decision-making ability and efficiency (Hsu 2019). However, strengthening system organizational management control and maintenance can reduce and eliminate the impact of human control and ensure effective implementation of subsidiary internal control (Kostova et al. 2015). Auditors can also use computer-aided audit technology to improve audit efficiency, discover subsidiary operating problems in a timely way, and achieve effective supervision (Fan 2020). In addition, the parent company can establish an internal management reporting system to comprehensively supervise the financial status, capital use, and business operations of subsidiaries, enhancing the timeliness and pertinence of internal management (Zhai et al. 2020).

### Integrated audit management

Internal audit can promote consistent strategic objectives and management systems in parent-subsidiary organizations, improve business performance, and ensure internal control compliance (Ackermann and Fourie 2013; Chang et al. 2018). An effective internal control system can properly regulate the company’s various operations and contribute greatly to its sustainable and stable operation (Aziz et al. 2017). The parent company may release subsidiary annual internal audit plans according to each subsidiary’s business characteristics and business scale and assign auditors to supervise the plan’s implementation (Elbardan et al. 2016). The parent company should regularly review subsidiary financial reports to evaluate their performance and strengthen supervision of the auditing process. Auditors in the parent company should conduct special audits on major business projects and investment activities of subsidiaries to evaluate the feasibility and benefits of investment projects (Weng and Cheng 2019). The internal audit department should classify the operational problems found in each audit, using the parent-subsidiary information exchange and feedback mechanism, reasonably determine the risk level, formulate detailed rectification plans, and track rectification to ensure the effectiveness of the internal audit (Ke et al. 2008).

## An innovative hybrid architecture

**Fig. 1:**
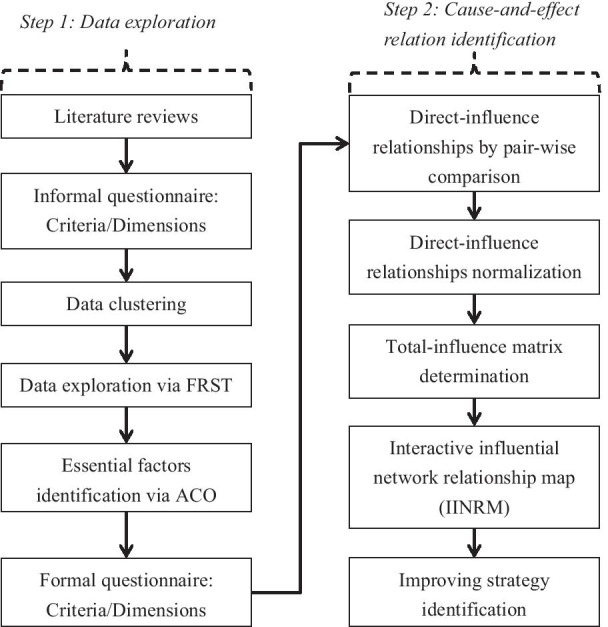
The flowchart of the introduced hybrid model

As of 2020, AI has demonstrated its usefulness in the modelling of linear and non-linear interpretations of a given dataset for over two decades and has shown incredible potential for learning the non-linear relationships among criteria by analyzing historical messages from experimental datasets (Okafor et al. 2020). Hybrid/integrated systems that focus on combining several different models’ outputs and translating them into a synthesized result are efficient at improving the predictive performance of a single system. In fact, hybrid/integrated systems have demonstrated amazing predictive qualities with superb capabilities for generalization without omitting local or specific knowledge (Zhang and Ma 2012; Zhou 2012). Their superior performance at predictive analysis has made them one of the best and most influential AI methods thus far (Fernández-Delgado et al. 2014; González et al. 2020; Wu et al. 2008). This finding is also echoed by West et al. (2005), who indicated that even a fraction of improvement in prediction quality can translate into a considerable financial savings. The hybrid/integrated architecture (see Fig. 1) of an internal control system for subsidiary supervision and management involves two steps: (1) data exploration via ACO-FRST and (2) cause-and-effect relation identification via DEMATEL. Each step is described as follows.

- Step 1: To construct the foundation of this research and realize what kinds of factors have actual and considerable impact on subsidiary supervision and management, we reviewed many related works to determine the relevant criteria/dimensions and to represent them in a hierarchical structure (i.e., informal questionnaire construction). However, data exploration is required, because too many criteria/dimensions will confuse decision makers and result in inappropriate judgments. FRST offers superior ability in handling data with imperfect messages. Therefore, we use FRST to deal with the data exploration task. Furthermore, as FRST belongs to a “supervised learning algorithm” that refers to a function from labeled training data consisting of a set of input–output pairs, we initially have to determine the FRST decision variable using a clustering algorithm. Unfortunately, minimal reduct determination for FRST is time-consuming due to its complicated calculation procedures. However, as this calculation procedure can be transformed into a combinational optimization task, we consider ACO with its many advantages at solving optimization tasks. The formal questionnaire is derived after going through all the mentioned procedures.
- Inspired by the hybrid/integrated system, the selected criteria/dimensions are then inserted into DEMATEL to gain much more in-depth insights. First, the direct-influence relationship must be decided by conducting a pairwise comparison. Normalization is taken to prevent an outcome with large variances among criteria/dimensions. Sequentially, we summarize all the rows and columns from a normalized direct-influence matrix to form a total-influence matrix. Finally, based on the information from a prior stage, IINRM can be derived, and users can realize which criterion/dimension has considerable influence on the work of subsidiary supervision and management. The detailed description of each methodology appears in the following Sect. 3.1.

### Ant colony optimization-based fuzzy rough set theory (ACO-FRST)

In most situations when the data from analyzed domains contain two different structures (i.e., crisp data and real-valued data), the traditional RST encounters a problem because it cannot handle the latter type of data. Thus, a tool must be developed to handle two different structures simultaneously. This goal can be achieved by FRST, which encapsulates the concept of vagueness from the fuzzy set theory (FST) and indiscernibility from RST. A brief illustration of FRST runs as follows (Jensen and Shen 2005; Wang et al. 2007).

The core of FRST is fuzzy crisp equivalence. Crisp equivalence classes can be extended by considering a fuzzy similarity relation $G$ on the universe, which describes the level of similarity of two elements in $G$. Relying on the fuzzy similarity relation, the fuzzy equivalence class $\left[ a \right]_{G}$ for an instance closest to $a$ can be represented as:

1 $$\mu_{{\left[ a \right]_{G} }} (b) = \mu_{G} (a,b)$$

The following axioms hold for a fuzzy equivalence class $F$ (Höhle 1988):

$$\begin{aligned} & \exists_{a} ,\mu_{F} (a) = 1 \\ & \mu_{F} (a) \wedge \mu_{G} (a,b) \le \mu_{F} (b) \\ & \mu_{F} (a) \wedge \mu_{F} (b) \le \mu_{G} (a,b) \\ \end{aligned}$$

The first axiom states that an equivalence class is non-empty. The next axiom represents that the elements in $b$’s neighborhood are in the equivalence class of $b$. The final axiom indicates that any two elements in $F$ are related by means of $S$. Equations (2) and (3) represent the fuzzy $Q$-lower and $Q$-upper approximations, respectively.

2 $$\mu_{{\underline{Q} A}} (F_{i} ) = inf_{a} \max \left\{ {1 - \mu_{{F_{i} }} (a),\mu_{A} (a)} \right\}\begin{array}{*{20}c} {} \\ \end{array} \forall i$$

3 $$\mu_{{\overline{Q} A}} (F_{i} ) = \sup_{a} \min \left\{ {\mu_{{F_{i} }} (a),\mu_{A} (a)} \right\}\begin{array}{*{20}c} {} \\ \end{array} \forall i$$

Here, $F$ represents a fuzzy equivalence class belonging to ${\mathbb{R}}/Q$ that in turn is designated as the partition of ${\mathbb{R}}$ for a given feature subset $Q$. For a feature $x$, the partition of the universe by $\left\{ x \right\}$ (represented as ${\mathbb{R}}/IND\left( {\left\{ x \right\}} \right)$) is taken as fuzzy equivalence classes for that feature. In the crisp case, ${\mathbb{R}}/Q$ contains sets of objects grouped together that are hard to discriminate by utilizing two features $x$ and $y$. In the fuzzy case, objects may belong to numerous dissimilar classes, and so two Cartesian products of ${\mathbb{R}}/IND\left( {\left\{ x \right\}} \right)$ and ${\mathbb{R}}/IND\left( {\left\{ y \right\}} \right)$ must to be taken into consideration for calculating ${\mathbb{R}}/Q$. In general, we have:

4 $${\mathbb{R}}/Q = \otimes \left\{ {x \in Q:{\mathbb{R}}/IND\left( {\left\{ x \right\}} \right)} \right\}$$

Although the universe of discourse in feature selection is finite, this is not the case in general. Therefore, $\sup$ and $\inf$ are considered in prior equations. The fuzzy lower and upper approximations can be restructured as follows:

5 $$\mu_{{\underline{Q} A}} (a) = \mathop {\sup }\limits_{{F \in {\mathbb{R}}/Q}} \min \left( {\mu_{F} \left( a \right),\begin{array}{*{20}c} {} \\ \end{array} \mathop {\inf }\limits_{{b \in {\mathbb{R}}}} \max \left\{ {1 - \mu_{F} \left( b \right),\mu_{A} \left( b \right)} \right\}} \right)$$

6 $$\mu_{{\overline{Q} A}} (a) = \mathop {\sup }\limits_{{F \in {\mathbb{R}}/P}} \min \left( {\mu_{F} \left( a \right),\begin{array}{*{20}c} {} \\ \end{array} \mathop {\sup }\limits_{{b \in {\mathbb{R}}}} \min \left( {\mu_{F} \left( a \right),\mu_{A} \left( b \right)} \right)} \right)$$

In practical application, not all $b \in {\mathbb{R}}$ are taken into consideration – only those where $\mu_{F} \left( b \right)$ is non-zero. FRST can be represented as the following tuple $\left\langle {\underline{Q} A,\overline{Q} A} \right\rangle$. Each set in ${\mathbb{R}}/Q$ represents an equivalence class. The level of an instance belonging to such an equivalence class is computed by performing the conjunction of constituent fuzzy equivalence classes $F_{i} ,i = 1, \ldots ,n.$

7 $$\mu_{{F_{1} \cap \cdots \cap F_{n} }} \left( a \right) = \min \left( {\mu_{{F_{1} }} \left( a \right),\mu_{{F_{2} }} \left( a \right), \ldots ,\mu_{{F_{n} }} \left( a \right)} \right)$$

By the extension of the crisp positive region in RST, the membership of an instance classified into the fuzzy positive region can be represented in Eq. (8).

8 $$\mu_{{POS_{Q} \left( P \right)}} (a) = \mathop {\sup }\limits_{{A \in {\mathbb{R}}/P}} \mu_{{\underline{Q} A}} (a)$$

Instance $a$ is not classified into the positive region only if the equivalence class it belongs to is not a constituent of the positive region. By performing the concept of the fuzzy positive region, the dependency function can be expressed in Eq. (9).

9 $$\gamma ^{\prime}_{Q} (P) = \frac{{\left| {\mu_{{POS_{Q} (p)}} (a)} \right|}}{{\left| {\mathbb{R}} \right|}} = \frac{{\sum\nolimits_{{a \in {\mathbb{R}}}} {\mu_{{POS_{Q} (P)}} (a)} }}{{\left| {\mathbb{R}} \right|}}$$

The minimal subset can be determined by performing Eq. (9) to gauge the quality of the selected subset (Yue 2019). This study considers one swarm intelligence method, called ant colony optimization (ACO), that has proven its usefulness in the optimization task (Cornelis et al. 2010; Jensen and Shen 2005). ACO is a simulation of the behavior of ants foraging. When food is discovered while foraging, as the ants transport it back and forth, their body secretes a special substance known as a pheromone. By spreading these odors, ants are able to tell other ants which direction they should follow to find the food source. This concept transforms rule extraction into an optimization problem and summarizes the knowledge that can be easily understood by the user in order to strengthen the decision maker’s judgment.

Equation (10) represents the probability that an ant moves from node *i* to node *j* at time *t*.

10 $$l_{ij}^{m} (t) = \frac{{\left[ {\alpha_{ij} (t)} \right]^{\beta } \cdot \left[ {\eta_{ij} } \right]^{\lambda } }}{{\sum\nolimits_{{p \in J_{i}^{m} }} {\left[ {\alpha_{ip} (t)} \right]^{\beta } \cdot \left[ {\eta_{ip} } \right]^{\lambda } } }}$$

Here, $m$ denotes the number of ants, $J_{i}^{m}$ represents the set of ant $m$’s unvisited node, $\eta_{ij}$ expresses the heuristic desirability of selecting node $j$ when at node $i$, and $\alpha_{ij} (t)$ indicates the amount of pheromone on edge $\left( {i,j} \right)$. Here, $\beta$ and $\lambda$ are determined manually. The pheromone on each edge can be updated by utilizing the following equation.

11 $$\alpha_{ij} (t + 1) = (1 - \rho ) \cdot \alpha_{ij} (t) + \Delta \alpha_{ij} (t)$$

Here, $$\Delta \alpha_{ij} (t) = \sum\limits_{m = 1}^{n} {\left( {\gamma ^{\prime}\left( {G^{m} } \right)/\left| {G^{m} } \right|} \right)}$$.

The term *ρ* represents the evaporation coefficient of the pheromone, and $G^{m}$ is the feature subset determined by ant $m$. The pheromones are updated based on the goodness of the feature subset ($\gamma ^{\prime}$) in FRST. More detailed illustrations of ACO-FRST can be seen in Cornelis et al. (2010) and Jensen and Shen (2005).

### DEMATEL

Initiated by Gabus and Fontela (1972), DEMATEL is a structural technique that analyzes interdependent relationships and models the cause-and-effect relationships between complex elements of a system. It has been widely and successfully applied to resolve real-life problems (Chen et al. 2015, 2018; Hu et al. 2020; Lin and Hsu 2018; Liou et al. 2016; Peng and Tzeng 2019; Petrovic and Kankaras 2020; Tzeng and Huang 2012). Given the cluster of intertwined issues in complex systems, DEMATEL is appropriate and suitable for dealing with such problems (Hu et al. 2018a, b). DEMATEL’s detailed procedure is summarized below.

**Step 1** Creating the initial direct-influence matrix (**Z**): A committee of H respondents is formed to rate the direct-influence relationship by pairwise comparison of any two elements, based on an integer scale from 0 to 4, where 0 indicates *no influence*” 1 represents *low influence,* 2 stands for *medium influence*, 3 indicates *high influence*, and 4 is *very high influence*. A non-negative $${\varvec{X}}^{h} = [x_{ij}^{h} ]_{n \times n} ,$$ matrix for each respondent is $${\varvec{X}}^{h} = [x_{ij}^{h} ]_{n \times n} ,$$$$1 \le h \le H,$$ where X^1^,…, X^h^, …, X^H^ are the answer matrices by the *H* respondents, and *n* is the number of elements in the system. The average matrix **Z** of all respondents can be constructed using Eq. (12).

12 $$\user2{Z = }\left[ {\begin{array}{*{20}l} {z_{11} } \hfill & \ldots \hfill & {z_{{1{\varvec{j}}}} } \hfill & \ldots \hfill & {z_{{1{\varvec{n}}}} } \hfill \\ \vdots \hfill & {} \hfill & \vdots \hfill & {} \hfill & \vdots \hfill \\ {z_{i1} } \hfill & \ldots \hfill & {z_{{{\varvec{ij}}}} } \hfill & \ldots \hfill & {z_{{{\varvec{in}}}} } \hfill \\ \vdots \hfill & {} \hfill & \vdots \hfill & {} \hfill & \vdots \hfill \\ {z_{{{\varvec{n}}1}} } \hfill & \ldots \hfill & {z_{{{\varvec{nj}}}} } \hfill & \ldots \hfill & {z_{{{\varvec{nn}}}} } \hfill \\ \end{array} } \right]$$

where *z*_*ij*_ is the average score of each indicator for each expert.

**Step 2** Normalizing the direct-relation average matrix (**D**): The normalized direct-relation average matrix is computed using Eqs. (13) and (14), and the values of the principal diagonal elements are all equal to zero (i.e., zero matrix, **Z**_ii_ = 0).

13 $${\varvec{B}} = \gamma \times {\varvec{Z}}$$

14 $$\gamma = \min \left\{ {\frac{1}{{\max_{1\; \le i \le n} \sum\nolimits_{j = 1}^{n} {z_{ij} } }},\frac{1}{{\max_{1\; \le j \le n} \sum\nolimits_{i = 1}^{n} {z_{ij} } }}} \right\}$$

**Step 3** Attaining the total-influence relation matrix (**T**): The total-influence relation matrix ${\varvec{T}} = \mathop {\lim }\limits_{h \to \infty } ({\varvec{B}} + {\varvec{B}}^{2} + \cdots + {\varvec{B}}^{h} ) = {\varvec{B}}({\varvec{I}} - {\varvec{B}})^{ - 1}$ ${\varvec{T}} = \mathop {\lim }\limits_{h \to \infty } ({\varvec{B}} + {\varvec{B}}^{2} + \cdots + {\varvec{B}}^{h} ) = {\varvec{B}}({\varvec{I}} - {\varvec{B}})^{ - 1}$ can be derived by summing all direct and indirect influences of components on each other, as shown in Eq. (15):

15 $${\varvec{T}} = \mathop {\lim }\limits_{h \to \infty } ({\varvec{B}} + {\varvec{B}}^{2} + \cdots + {\varvec{B}}^{h} ) = {\varvec{B}}({\varvec{I}} - {\varvec{B}})^{ - 1}$$

where ***I*** is an identity matrix.

**Step 4** Developing the interactive influential network relationship map (IINRM): The vectors of the total influence matrix **T** are the sum of rows (*R*) and sum of columns (*S*), as shown in Eqs. (16) and (17).

16 $$R = (r_{i} )_{n \times 1} = \left[ {\sum\nolimits_{j = 1}^{n} {t_{ij} } } \right]_{n \times 1} = (r_{1} , \ldots ,r_{i} , \ldots ,r_{n} )^{^{\prime}}$$

17 $$S = (s_{j} )_{n \times 1} = \left[ {\sum\nolimits_{i = 1}^{n} {t_{ij} } } \right]^{^{\prime}}_{1 \times n} = (s_{1} , \ldots ,s_{j} , \ldots ,s_{n} )^{^{\prime}}$$

where $r_{i}^{D/C}$ denotes the row sum $(r_{i}^{C} + s_{j}^{C} )$ of all direct and indirect influences from each dimension/criterion $(r_{i}^{C} + s_{j}^{C} )$ on all other dimensions *(D)*/criteria *(C)* and is called the degree of influence impact. By contrast, $(r_{i}^{C} + s_{j}^{C} )$ represents the column sum $(r_{i}^{C} + s_{j}^{C} )$ of both direct and indirect effects by dimension/criterion *j* from other dimensions/criteria (factors) and is called the degree of influenced impact. When $(r_{i}^{C} + s_{j}^{C} )$, the sum $(r_{i}^{C} + s_{j}^{C} )$ is a measure of the degree of importance of factor (criterion) *i* in the whole system. The difference $(r_{i}^{C} - s_{j}^{C} )$ divides the criteria into the cause group (positive) and the effect group (negative). Specifically, if $r_{i}^{C(D)} - s_{j}^{C(D)} > 0$, then the criterion/dimension $r_{i}^{C(D)} - s_{j}^{C(D)} < 0$ is considered a “cause” factor, while if $r_{i}^{C(D)} - s_{j}^{C(D)} < 0$, then the criterion/dimension $r_{i}^{C(D)} - s_{j}^{C(D)} < 0$ is considered an “effect” factor. An IINRM constructed based on the total influence relation matrix **T** can be derived by mapping the dataset of $(r_{i}^{C(D)} + s_{j}^{C(D)} ,r_{i}^{C(D)} - s_{j}^{C(D)} )$, which fully illustrates the structure relations of components.

## Empirical results

This section explains the process of data collection and questionnaire design, and conducts the empirical analysis using the DEMATEL technique based on the results of expert opinions.

### Data collection and questionnaire design

**Fig. 2:**
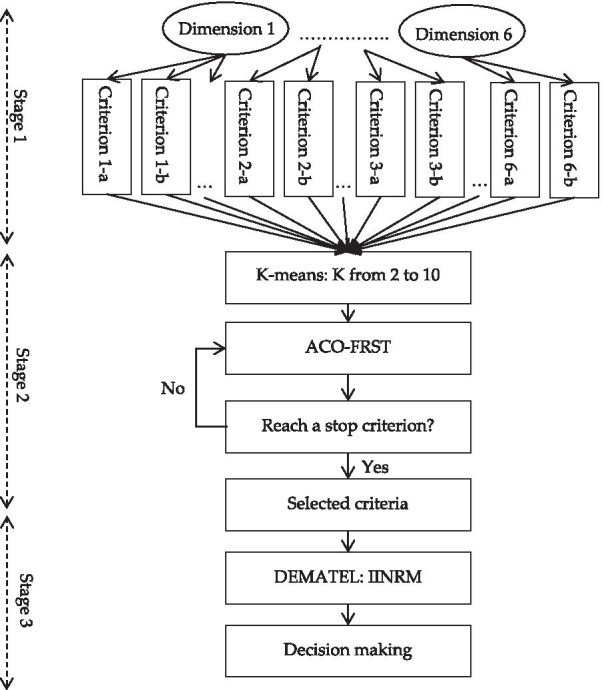
The architecture of a three-stage questionnaire design

This study adopts a three-stage questionnaire design (see Fig. 2), involving dimensions and criteria, a pre-test questionnaire, and a formal questionnaire. In the first stage, six dimensions and 31 criteria were determined based on the literature and professional in-depth reviews by experts with extensive experience, as shown in Table 1. In the second stage, a pre-test questionnaire was created based on the 31 criteria in Table 1. The pre-test questionnaire survey was responded to by 10 heads of internal audit departments at companies in Guangzhou and Shanghai. The domain experts were asked to score the impact of the 31 criteria in the pre-test questionnaire on subsidiary supervision and management using a scale from 0 (extremely unimportant) to 10 (extremely important). However, providing a specific number on the degree of influence between criteria by experts is not an easy and intuitive job. Due to the increasing difficulties of decision analysis, experts are familiar with the confidence level in their assessment (Si et al. 2017; Ding and Liu 2018). To combat this and gain more insights, the confidence interval is incorporated into a 10-point scale.

**Fig. 3:**
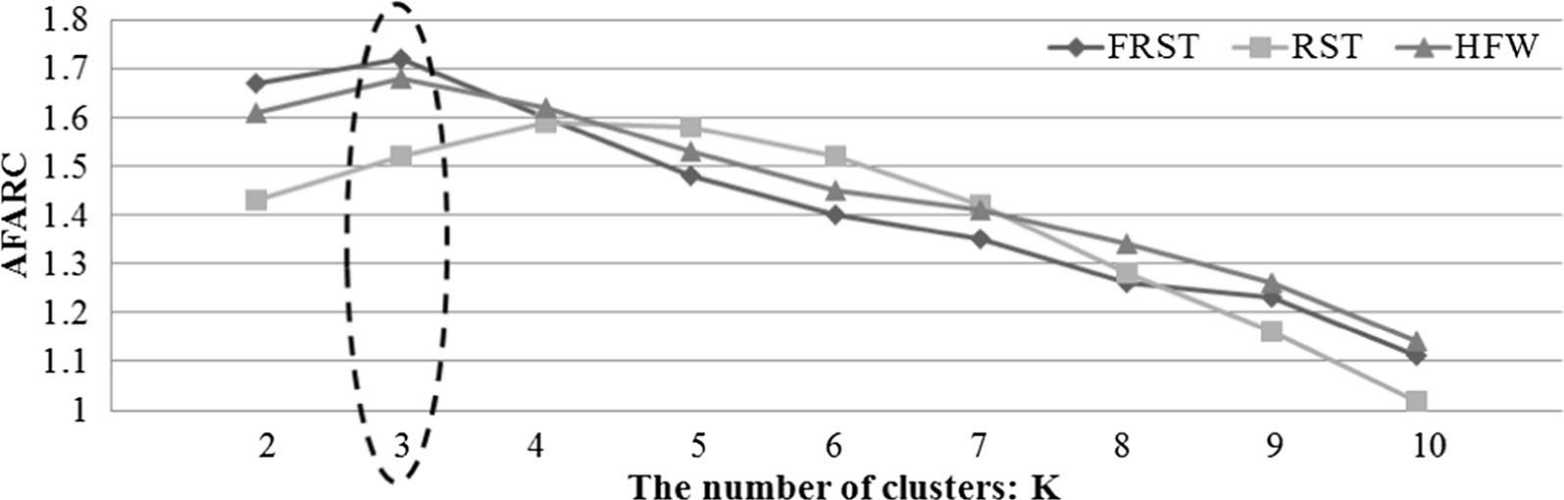
The results of comparisons

ACO-FRST belongs to a supervised learning technique. Before performing ACO-FRST to determine the most important criteria, the decision variable should be decided in advance. In accordance with the work done by Thangavel et al. (2005), we perform K-means to determine the decision variable. Here, k is set from 2 to 10, and the aggregation of forecasting accuracy and rule coverage (i.e., AFARC) is taken as an evaluation judgment. To confirm the effectiveness of FRST, the other two data exploration techniques are considered: classical RST (Pawlak 1982), and hybrid filter-wrapper subset selection (HFW) (Lin and Hsu 2017). The result (see Fig. 3) states that FRST outperforms the other two techniques. To prevent the result merely happening by coincidence, we apply the Freidman test (one of the non-parametric statistical tests), which is performed to test the differences between groups when the dependent attribute being assessed in ordinal. Apart from previous studies that just consider one assessment measure (i.e., accuracy), this study further takes the other two measures (precision and recall) into consideration so as to reach a reliable outcome. Table 2 displays the results. We can see that FRST performs better than the other two models under all assessment measures. A description of each criterion derived from FRST is represented in Tables 3 and 4.

**Table 2:** The compared results

The number of clusters: K = 3 (rank)			
	Accuracy	Precision	Recall
FRST	85.00 (1)	86.67 (1)	83.86 (1)
RST	70.56 (3)	72.22 (3)	69.72 (3)
HFW	74.12 (2)	82.22 (2)	68.56 (2)
p-value	0.022**	0.042**	0.022**

^*^, **, ***p < 0.1, 0.05, 0.001, respectively

**Table 3:** The selected criteria by ACO-FRST

Status	Selected criteria	Forecasting accuracy	Rule coverage	AFARC
K = 2	a2, a3, b1, b3, b4, c1, c4, c5, d1, d4, e3, e4, f1, f3, f5, f8	0.82	0.85	1.67
***K = 3***	***a1, a2, b1, b2, c1, c2, c3, d1, d2, d3, e1, e2, f1, f2, f3, f4***	***0.85***	***0.87***	***1.72***
K = 4	a1, a3, a4, b1, b3, c1, c3, c4, c5, d1, d3, d4, e1, e2, e3, f1, f2, f3,f6	0.78	0.82	1.6
K = 5	a1, b3, b4, c1, c3, c4, d1, d2, d5, e3, f1, f5, f8	0.74	0.74	1.48
K = 6	a2, b1, b2, c1, c4, c6, d1, d4, d5, e1, e2, f1, f3, f4, f8	0.71	0.69	1.4
K = 7	a1, b2, b4, c1, c3, c6, d3, d5, e1, e3, f2, f4, f7, f8	0.68	0.67	1.35
K = 8	a2, b1, b4, c3, c5, c6, d2, d4, e3, e4, f1, f4, f7	0.64	0.62	1.26
K = 9	a1, b2, c2, c3, c6, d1, d3, e4, f2, f6, f8	0.62	0.61	1.23
K = 10	a1, b1, b3, c1, c6, d2, d4, d5, e4, f1, f7, f8	0.53	0.58	1.11

**Table 4:** Supervision and management of subsidiaries factor (criterion) assessment architecture

Dimensions	Criteria	Descriptions	Sources
A	Directors and supervisors of subsidiaries ($a_{1}$)	The power of the parent company to appoint and remove directors and supervisors of the subsidiaries	Situmoranga and Japutra (2019), Cai et al. (2018)
	Department division of responsibilities in subsidiaries ($a_{2}$)	Clarity of the division of authority and responsibility between departments in subsidiary companies	Boussebaa (2015)
B	Management of subsidiary operating efficiency ($b_{1}$)	The extent to which subsidiaries completes the expected performance indicators (e.g. sales, profits, inventory, etc.) of the parent company	Zhai et al. (2020)
	Implementation of the subsidiaries' annual business plan ($b_{2}$)	Subsidiaries complete the business objectives and business plans issued by the parent company during each year	Matolcsy and Wakefield (2017)
C	Management of budget and final accounts ($c_{1}$)	Annual budget and final accounts management issued by the parent company to subsidiaries	Dunk 2001, Defranco and Schmidgall (2017)
	Payment management ($c_{2}$)	Management of accounts receivable, payable, etc	Sonia et al. (2014)
	Purchasing and supply management ($c_{3}$)	Management of the organization, implementation and control of the procurement process of subsidiaries	Weele and Raaij (2014), Johnsen (2018)
D	Operations management ($d_{1}$)	To plan, organize, implement and control the business process of the enterprise, the essence of which is to manage financial accounting, technology, production and operation, marketing and human resources management in an integrated manner	Bromiley and Rau (2015)
	Investment management ($d_{2}$)	Parent company's management of the quality (return on assets) and efficiency (payback period) in subsidiaries’ investments	Hsu and Liu (2018)
	Capital Management ($d_{3}$)	Capital management mainly includes centralized management of fund budget, cash, settlement and financing	Mortensen (2014)
E	Financial and business communication system ($e_{1}$)	Based on the communication of business and financial information, parent company adopts control means to accurately grasp the actual operating conditions of subsidiaries and push subsidiaries to achieve organizational goals	Poston and Grabski (2015)
	Provision of management reports ($e_{2}$)	Subsidiaries provides the parent company with various internal management reports(including fund analysis reports, operating financial activities reports, asset use reports, investment benefit analysis reports, internal audit reports, etc..)	Chang and Taylor (1999)
F	Construction of internal control system in subsidiaries ($f_{1}$)	Risk management control, operational level control, major investment project control, internal audit system, reporting and disclosure control, financial statement management, etc.	Aziz et al. (2017)
	Implementation of the annual internal audit plan ($f_{2}$)	The parent company issues the subsidiaries’ internal audit plan each year according to the operating characteristics and business scale of each subsidiary, and assigning the corresponding auditor to evaluate the implementation of subsidiaries’ internal audit plan	Chen et al. (2015), Hu et al. (2018a, b)
	Project audits of subsidiaries ($f_{3}$)	Special audit of major business projects and investment activities of subsidiaries to judge the feasibility of investment projects and the efficiency of capital use	Weng and Cheng (2019)
	Rectification of internal audit issues ($f_{4}$)	The audit department classifies the operational problems found in each audit, using the information exchange and feedback mechanism in parent-subsidiary companies, reasonably determining the risk level, and formulating a detailed rectification plan (clear rectification requirements, time limit, responsible person, result), follows up and evaluates the internal rectification situation	Ke (2018)

^*^A represents organizational control structure; B represents business strategy management; C represents construction of a management system; D represents major financial management; E represents business and financial information system management; and F represents integrated audit management

In the third stage, the formal questionnaire was constructed according to Table 4 and the results of the expert knowledge and was administered. A total of 20 interviews were conducted with chief audit executives or heads of internal audit from China’s listed companies in Guangzhou, Shenzhen, and Shanghai. Each questionnaire was conducted through face-to-face survey interviews of more than 1.5 h between January 2019 and May 2019. The respondents were requested to make pair-comparison judgements of 16 criteria and the satisfaction of criteria at this stage. The estimation of the impact of a pair-comparison was based on scoring using a five-point scale (0 = *absolutely no influence* and 4 = *very high influence*) based on the opinions/perceptions gathered from the domain experts. To determine the reliability of the sample collection, a random selection of 19 questionnaires was used to capture the consensus level with an average gap ratio of 1.01% < 5% (i.e., more than 95% confidence), indicating consensus (see Note in Table 5). Additionally, the performance questionnaire invited respondents to rate from 0 = *extreme dissatisfaction* to 10 = *extreme satisfaction*. Consequently, 20 completed expert forecasting questionnaires serve as the basis for the empirical analysis of the methodology described in this study.

**Table 5:** Sum of cause $r_{i}$ and effect $s_{i}$ influence among the core dimensions and criteria

Dimensions/criteria	Row sum ($r_{i}$)	Column sum ($s_{i}$)	$r_{i} + s_{i}$	$r_{i} - s_{i}$
Organizational control structure (A)	0.707	0.467	1.174	0.240
Directors and supervisors of subsidiaries ($a_{1}$)	0.218	0.130	0.348	0.088
Department division of responsibilities in subsidiaries ($a_{2}$)	0.101	0.189	0.290	-0.088
Business strategy management (B)	0.514	0.494	1.007	0.021
Management of subsidiary operating efficiency ($b_{1}$)	0.154	0.106	0.260	0.048
Implementation of the subsidiaries' annual business plan ($b_{2}$)	0.095	0.143	0.238	-0.048
Construction of a management system (C)	0.501	0.494	0.995	0.007
Management of budget and final accounts ($c_{1}$)	0.170	0.202	0.372	-0.032
Payment management ($c_{2}$)	0.164	0.202	0.366	-0.038
Purchasing and supply management ($c_{3}$)	0.243	0.173	0.416	0.070
Major financial management (D)	0.514	0.473	0.987	0.041
Operations management ($d_{1}$)	0.228	0.151	0.379	0.077
Investment management ($d_{2}$)	0.153	0.205	0.358	-0.052
Capital Management ($d_{3}$)	0.178	0.204	0.382	-0.026
Business and financial information system management (E)	0.611	0.445	1.056	0.166
Financial and business communication system ($e_{1}$)	0.171	0.092	0.263	0.079
Provision of management reports ($e_{2}$)	0.105	0.129	0.234	-0.024
Integrated audit management (F)	0.509	0.718	1.227	-0.209
Construction of internal control system of subsidiaries ($f_{1}$)	0.254	0.256	0.510	-0.002
Implementation of the annual internal audit plan ($f_{2}$)	0.233	0.298	0.531	-0.065
Project audits of subsidiaries ($f_{3}$)	0.295	0.225	0.520	0.070
Rectification of Internal audit issues ($f_{4}$)	0.286	0.289	0.575	-0.003

Average gap ratio $= \frac{1}{n \times (n - 1)}\sum\nolimits_{i = 1}^{n} {} \sum\nolimits_{j = 1}^{n} {({{\left| {\overline{z}_{ij}^{20} - \overline{z}_{ij}^{19} } \right|} \mathord{\left/ {\vphantom {{\left| {\overline{z}_{ij}^{20} - \overline{z}_{ij}^{19} } \right|} {\overline{z}_{ij}^{20} }}} \right. \kern-\nulldelimiterspace} {\overline{z}_{ij}^{20} }}} ) \times 100\% = 1.01\% < 5\%$, *n* = 16 is number of key factors. This result indicates that significant confidence of consensus is 98.99%, where $\overline{z}_{ij}^{19}$ and $\overline{z}_{ij}^{20}$ are the average scores of the domain experts for 19 and 20, respectively.

### Creation of an IINRM using DEMATEL

Table 5 shows the results of DEMATEL’s calculations. Of the six dimensions, dimension A (organizational control structure, $r_{i} - s_{i} = 0.{24}0$) is the most influential factor, which means that dimension A had the strongest impact and its improvement will lead to improvement in other dimensions. Dimension C (construction of a management system, $r_{i} - s_{i} = 0.00{7}$) and dimension F (integrated audit management, $r_{i} - s_{i} = - 0.{2}0{9}$) are the least important factors. Organizational control structure (A), business strategy management (B), construction of a management system (C), major financial management (D), and business and financial information system management (E) are positive, which confirms their direct effect on other dimensions. On the other hand, the $r_{i} - s_{i}$ value of integrated audit management (F) is negative, which means this dimension is influenced by other dimensions. In the criteria assessment of a subsidiary supervision framework, criterion *a*_*1*_ (directors and supervisors of subsidiaries) has the largest $r_{i} - s_{i}$ value ($r_{i} - s_{i} = 0.0{88}$) among all the criteria, indicating that this criterion has the largest impact on other criteria. However, with a minimal value of − 0.088, criterion *a*_*2*_ (department division of responsibilities in subsidiaries) is most easily influenced by other criteria.

**Fig. 4:**
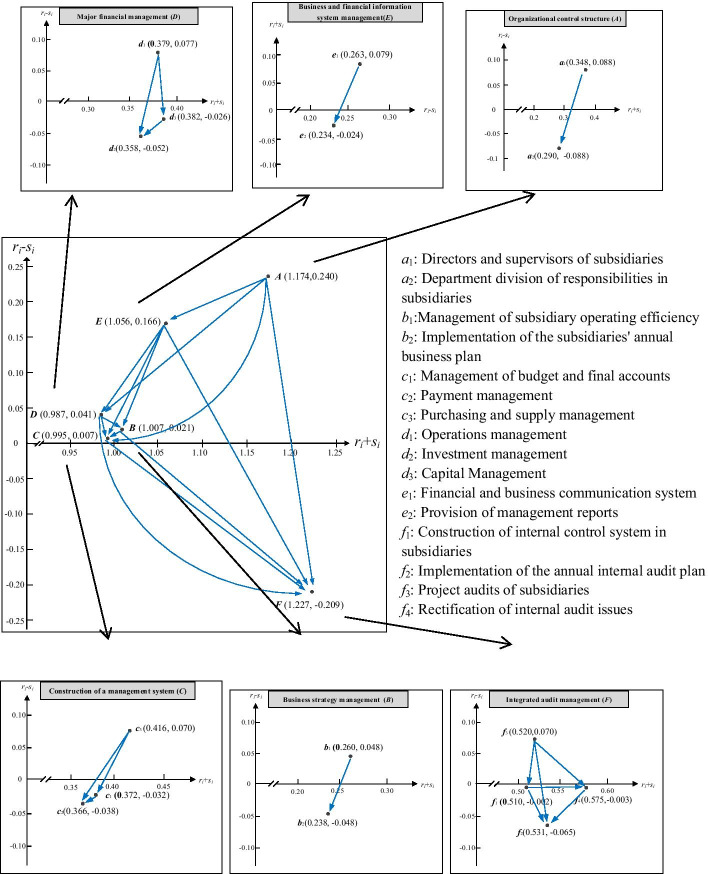
The IINRM of influence relationships based on DEMATEL within supervision and management of subsidiaries

The IINRM in this study was constructed by measuring the degree of interaction between the six dimensions and 16 criteria of a parent-subsidiary supervision framework using the DEMATEL method, as shown in Fig. 4. The horizontal axis $r_{i} + s_{i}$ and the vertical axis $r_{i} - s_{i}$ represent the degree of a relationship between criteria and the degree of causality between variables, respectively. IINRM enables us to clearly recognize the interdependence of various criteria in the parent-subsidiary supervision framework. For example, dimension A (organizational control structure) acknowledges that it has a direct impact on other dimensions, such as dimension E (business and financial information system management) and also has a significant influence on dimension B (business strategy management), dimension C (construction of a management system), dimension D (major financial management), and dimension F (integrated audit management). Dimension F (integrated audit management) was below the horizontal axis, indicating it is the most susceptible to other dimensions.

## Discussion, implications, and practical application

This study analyzed the causal relationship between the dimensions and criteria in subsidiary supervision and management, based on the experience and knowledge of experts in business circles. The IINRM was then constructed using the DEMATEL method, as shown in Fig. 4. According to the results of the influence relationships, the priority of improving each dimension is: dimension A (organizational control structure), dimension E (business and financial information system management), dimension D (major financial management), dimension B (business strategy management), dimension C (construction of a management system), and dimension F (integrated audit management).

The results show that dimension A has the most critical and direct influence on the other dimensions. Therefore, dimension A (organizational control structure) should be given priority for improvement in the parent-subsidiary supervision process, as it will significantly impact the effect of supervision. According to “The Company Law of the People’s Republic of China,” subsidiaries are required to set up an organizational structure in accordance with the law to facilitate the parent company’s strategic coordination and management and control. Also, Birkinshaw (2008) finds that interaction between the directors and executives in the parent company and the managers in subsidiaries can promote the realization of organizational goals and the effect of quality supervision. Pudelko and Tenzer (2013) suggest that one of the ways a parent company can realize subsidiary supervision and control is to dispatch personnel to the subsidiaries’ key management positions. A scientific and reasonable organizational structure can guarantee enrichment of the effect of supervision. The parent company, as an investor, actively participates in the strategic decision-making of its subsidiaries by recommending directors and senior executives to the subsidiaries as a means of obtaining various types of information. In addition, criterion *a*_*1*_ (directors and supervisors of subsidiaries), criterion *b*_*1*_ (management of subsidiary operating efficiency), criterion *c*_*3*_ (purchasing and supply management), criterion *d*_*1*_ (operations management), criterion *e*_*1*_ (financial and business communication system), and criterion *f*_*3*_ (project audits of subsidiaries) each had a significant influence on each dimension. These six indicators (factors) are key factors in the supervision system and they significantly impact the effect of supervision in a parent-subsidiary company.

Among all the sub-indicators, directors and supervisors of subsidiaries (*a*_*1*_) has the highest influence on the other criteria. The board of directors is the core of corporate governance as well as the main body leading the company’s operations and strategic implementation measures. The governance level of the board of directors directly affects the company’s operating efficiency. The subsidiary’s board of directors is the authority that conducts comprehensive supervision and compliance management on behalf of the parent company (Kiel et al. 2010). Therefore, to strengthen subsidiary management and safeguard the legitimate rights and interests of the parent company as a funder, the parent company should constantly standardize and strengthen management of the duties and procedures of subsidiary directors and supervisors, who are appointed or recommended by the parent, and then improve the parent company’s management system and achieve coordinated development. Cai et al. (2018) found that a parent company’s supervision of the board of directors helps reduce the operating risks of its subsidiaries, and, as the parent company’s governance improves, managers in subsidiaries will more diligently perform their duties of disclosure and reporting. Du et al. (2015) also found that an active board of directors is a control mechanism for managing subsidiaries and a tool for responding to the external environment; the parent company should emphasize the assignment of the board of directors of its subsidiaries when conducting supervision. A financial and business communication system (*e*_*1*_) is also a valuable supervisory indicator. Whether the financial information in parent-subsidiaries is unblocked is related to the operational efficiency of the entire financial control system. An effective financial information control system creates realistic conditions for improving the effectiveness of financial information. The parent company can use the financial information system to understand the business activities of its subsidiaries in a timely manner and to provide timely evaluation and control of the financial and operational risks to improve the efficiency of supervision (Chen et al. 2015).

From the nature of a multinational company, the parent company should effectively conduct supervision over subsidiaries’ businesses, commercial channels, and customer resources. A special supervision and guidance department can be established in the parent company to maintain its overall benefits (Wang 2012). Despite the increasingly complex global environment, a close-knit linkage exists between the parent firm and its subsidiaries. The parent company is able to control the board of directors of the subsidiary and has the right to replace its directors and supervisors. Subsidiary firms need to communicate and implement the parent company’s relevant strategies and plans that are given to the subsidiaries. The parent company needs each subsidiary to provide effective feedback on market information, give suitable deployment for the latest development direction, share information, and have some discussion so as to fully grasp the operation status of each subsidiary. Good internal control and corporate governance tend to help form a predominant organization structure (Hsu and Liu 2018). Therefore, the empirical results of this paper provide a practical reference for the formulation of a multinational corporate’s internal supervision strategy, internal control, and internal audit of its subsidiaries.

The managerial implications of this proposed hybrid architecture are highlighted as follows. (1) A data exploration technique (i.e., ACO-FRST) is applied to determine the critical features from large datasets that still maintain the predictive capability of the model and to speed up the data processing procedure. In a vague and uncertain information environment, such as cross-border and cross-region supervision and management issues, the model demonstrates better superiority. (2) The selected factors via ACO-FRST are imported into DEMATEL to determine the most influential dimension and criteria. By doing so, the dependency and feedback relationship among key factors for subsidiary supervision and management can be clearly represented to respond to an ever-changing dynamic environment, especially in the context of today’s dramatic economic downturns.

## Conclusion and future research

This research contributes to the current literature in several ways. First, we propose an innovative hybrid technique to resolve the problem of unifying subsidiary supervision and management, linking FST and RST to provide more intrinsic information for internal audits. Second, we add to the stream of financial research that concentrates on internal control architecture development. Compared to other studies (i.e., information system adoption and financial trouble forecasting), works on the key factor extraction for subsidiary supervision and management are quite scarce. Third, the key factors screened by FRST are then fed into DEMATEL to depict the interrelated and intertwined relations among adopted criteria and to further examine their impact on the final decision. Fourth, we adopt interactive influential network relationship map (IINRM) derived from DEMATEL to realize which part of subsidiary supervision and management has to be modified first to receive the most effective response. Managers can thus consider the potential implications of this study to formulate future firm policy to reach the goal of sustainable development.

We propose an innovative hybrid technique for resolving the problem of unifying subsidiary supervision and management. ACO-FRST and DEMATEL have been chosen herein to explore the pattern and critical factors for subsidiary supervision and management. Based on the outcome derived from DEMATEL, the IINRM can be reached. IINRM determines the directions and ways that the indicators interact amongst themselves, which can help managers understand the underlying issues of subsidiary supervision and develop appropriate improvement strategies. Regarding business dimensions, the findings are based on expert knowledge, and the priorities for improvement include organizational control structure, business and financial information system management, major financial management, business strategy management, construction of a management system, and integrated audit management. Among all the criteria, “directors and supervisors of subsidiaries” represent the core of corporate governance and internal control for multinational corporations as well as the principal body for capturing a company’s operations and strategic implementation policies.

Although we successfully constructed a practical evaluation model for a transnational corporation, some other interesting views are worth examining for future work. The assessment architecture proposed herein is based on general guidelines, and other special circumstances can be considered, such as the impact of the recent COVID-19 pandemic or advanced quality of service mechanism on the supervision of subsidiaries. The adoption of more samples or other bio-inspired meta-heuristic algorithms, such as discrete antlion optimization approach (Barma et al. 2019), will improve the reliability and robustness of empirical results. Other multi-criteria group decision making approaches, such as Pythagorean m-polar fuzzy soft sets (Riaz and Tehrim 2020a, b, c), may have greater flexibility to handle data with imprecise messages and identify specific directions for subsidiary supervision and management.

Other multi-criteria group decision making (GDM) approaches, such as Pythagorean m-polar fuzzy soft sets (Riaz and Tehrim 2020a, b, c), the nearest consistent metrics (NCM)-based GDM (Lin et al. 2020a), the soft consensus-based GDM (Zhang et al. 2019), minimum adjustment-based GDM (Zhang et al. 2020), non-cooperative-based GDM (Chao et al. 2021) and more sophisticated algorithms for individual judgments aggregation (Kou et al. 2014; Lin et al. 2020b), may have greater flexibility to handle data with imprecise messages and also identify specific directions for subsidiary supervision and management.

## Data Availability

The datasets used during the current study are available from the corresponding author on reasonable request.
